# The Application of ^1^H Nuclear Magnetic Resonance (NMR), Gas Chromatography (GC) and Ultraviolet–Visible (UV-Vis) Spectroscopy Techniques to the Analysis of the Fatty Acid Profile as Quality of Argan Oil

**DOI:** 10.3390/ijms26115322

**Published:** 2025-06-01

**Authors:** Patrycja Słomczyńska, Paweł Siudem, Agnieszka Białek, Sławomir Kaźmierski, Katarzyna Paradowska

**Affiliations:** 1 Department of Organic and Physical Chemistry, Faculty of Pharmacy, Medical University of Warsaw, Banacha 1, 02-097 Warsaw, Poland; patrycja.slomczynska13@gmail.com; 2 School of Health and Medical Sciences, University of Economics and Human Sciences in Warsaw, 01-043 Warsaw, Poland; a.bialek@ifzz.pl; 3 The Kielanowski Institute of Animal Physiology and Nutrition, Polish Academy of Sciences, 05-110 Jabłonna, Poland; 4 Centre of Molecular and Macromolecular Studies, Polish Academy of Sciences, Sienkiewicza 112, 90-363 Łódź, Poland; slawomir.kazmierski@cbmm.lodz.pl

**Keywords:** argan oil, ^1^H NMR, gas chromatography, ultraviolet–visible spectroscopy, fatty acids, adulterations

## Abstract

This study utilised the fatty acid (FA) profiles of cosmetic argan oils from various producers obtained from retail outlets in Poland, Turkey and Morocco between November 2022 and November 2023 as an indicator to control the quality (i.e., purity) and origin (i.e., geographical origin) of the oils. The fatty acid profile was analysed using gas chromatography (GC), which revealed that the most prevalent fatty acid in argan oil is oleic acid (C18:1), followed by linoleic acid (C18:2) and, in order, palmitic acid (C16:0). Furthermore, the ^1^H NMR spectroscopy method was found to be both rapid and precise in identifying characteristic signals indicative of the presence of individual components (fatty acids) in argan oil, without the necessity for additional analyte processing. To analyse the results obtained, a PCA analysis was performed to discriminate between seven purified argan oil samples. Our study demonstrates the feasibility of implementing certain variables as authenticity and quality criteria. In the context of argan oils, the incorporation of limits for trans fatty acids and the capacity to discern origin through fatty acid profiling may prove to be of paramount importance. The results obtained demonstrated highly significant discrimination of five groups by region and three groups by preparation.

## 1. Introduction

The argan tree (*Argania spinosa*) is a member of the *Sapotaceae* family and is endemic to the Sus region of south-western Morocco [1]. In 1998, the argan tree was included in the UNESCO (United Nations Educational, Scientific and Cultural Organisation) list of protected biosphere elements [2]. The argan tree plays an important socio-economic role due to the versatility of its use and the raw materials derived from it [3]. For hundreds of years, the people of Morocco have utilised various parts of this plant for medicinal, cosmetic and dietary purposes. The seeds obtained from the fruit of the tree consist of approximately 58% oil, which is still used in traditional medicine and in pharmaceutical and cosmetic preparations [4].

Argan oil is a source of numerous valuable active ingredients, with an estimated 100+ different active substances present [5,6]. Firstly, it is composed of fatty acids, with an unsaturated/saturated ratio of 80:20 [3,7]. The composition is predominantly C18:2(9Z,12Z) (linoleic, LA) and C18:1(9Z) (oleic acids), with a lesser proportion of C18:3(9Z,12Z,15Z) (α-linolenic acid, ALA). Linoleic acid has been identified as a key component in the therapeutic efficacy of this oil in the treatment of dermatological inflammation and irritation [8]. Oleic acid is responsible for facilitating the penetration of active ingredients into the deeper layers of the skin [2]. EFAs (essential fatty acids) stimulate cellular activity, promoting the regeneration of tissue and the fading of scars and discolouration caused by conditions such as eczema, acne and burns [9]. Additionally, saturated fatty acids are present in smaller amounts, including C18:0 (stearic acid) (4–7%) and C16:0 (palmitic acid) (11–15%), which play a role in chronic inflammatory processes [2].

Furthermore, argan oil is rich in polyphenols and tocopherols, which are responsible for the oil’s antioxidant properties. Tocopherols constitute a group of plant antioxidants that occur in four forms: α-, β-, γ- and δ-. α-Tocopherol is the most prevalent form [10,11]. Nevertheless, it is estimated that γ-tocopherol constitutes the majority of the argan oil content (69%), which has been demonstrated to exhibit superior nitrogen-free radical scavenging properties and the capacity to impede cancer cell proliferation [2]. Vitamin E serves to safeguard against the oxidation of lipids present within cell membranes and to moisturise the epidermis. Additionally, it facilitates the formation of collagen and elastin [12].

Additionally, the oil contains squalene (0.3%, C_30_H_50_), the sole naturally occurring unsaturated hydrocarbon present in human sebum. When applied topically, it helps restore the epidermal hydrolipid barrier and replenishes missing lipid elements in the skin [2,9]. The oil softens and lubricates the skin, while also exhibiting antifungal and antibacterial effects [13]. The composition of argan oil is responsible for a number of beneficial effects, including antibacterial, antifungal, antioxidant, anti-inflammatory, anti-diabetic, anti-cancer, regenerative and moisturizing effects [1,4].

Argan oil is employed primarily in the cosmetic and pharmaceutical industries, and to a lesser extent in the food industry [6]. When used as an ingredient in cosmetics, argan oil is intended for application to allergic, mature, dry, sensitive and acne-prone skin [5]. It is used in anti-ageing preparations due to its strong antiradical and regenerative effects, which are attributed to its high content of tocopherols and carotenoids [5,6].

The production of argan oil is a labour-intensive and challenging process due to the limited area from which this raw material can be sourced [4]. Due to its scarcity, the pharmacological and nutritional properties of argan oil have resulted in its status as a luxury raw material with global appeal. The restricted production of the oil results in the adulteration of its composition by producers for financial gain. The adulteration of the fatty acid composition results in alterations to the physical and chemical properties of the oil, which consequently impact the nutritional effects of argan oil. The increasing frequency of composition adulteration underscores the necessity for rigorous authentication of the oil composition [14]. A variety of methods are being sought that can unambiguously determine the authenticity of the oil. For this purpose, methodologies for authenticating argan oil are being employed, which involve the determination of chemical markers by chromatographic methods, as well as DNA markers. The determination of the fatty acid profile can also be used to ascertain the geographical origin of the plant from which the oil was extracted, as well as to confirm its authenticity [15]. A large group of methods used in this type of research are spectroscopic methods. Numerous published studies have used FTIR to assess the authenticity or origin of oil [16,17]. In recent years, the use of the NMR method in oil research has also gained popularity. In the case of argan oil, however, the number of studies using NMR is small and is mainly limited to the use of low field (such as benchtop) [4]. Therefore, in our work, we undertook to apply and compare the results from FTIR, GC and NMR analysis using high field.

The objective of the present study was to analyse argan oil extracted from different sources and identify a marker to assess its quality. This was to be achieved using spectroscopic and/or chromatographic techniques (as a fingerprinting method), with the aim of generating a comprehensive and characteristic chemical profile of a given plant product and identifying a marker to assess quality.

## 2. Results and Discussion

The study material comprised products containing argan oil, which were defined and classified as cosmetic products. Seven products were selected for the study, which, according to the manufacturers, contained only 100% argan oil. Virgin argan oil intended for consumption and cosmetic applications consists mainly of acyl glycerides, accounting for nearly 99%.

### 2.1. GC-MS Determination of Fatty Acids

Gas chromatography coupled with mass spectrometry (GC-MS) is widely regarded as the gold standard for the identification and quantification of fatty acids [18]. Due to its properties, this method is able to identify non-volatile fatty acids present in a material after derivatization as fatty acid methyl esters (FAMEs) [19]. The GC-MS method employed in this study enabled the identification and quantification of 18 fatty acids. Analysis of the results made it possible to determine the fatty acids assigned to each oil. According to the literature, argan oil is 80% unsaturated fatty acids (78%) and saturated fatty acids (14%). The predominant fatty acids in argan oil are oleic acid (C18: 1) and linoleic acid (C18:2), accounting for 43–49% and 29–36% of the total fatty acid composition, respectively [20]. Oleic acid, classified as a monounsaturated fatty acid, belongs to the omega-9 family, while linoleic acid, a polyunsaturated fatty acid, is categorised as part of the omega-6 family. Palmitic and stearic acids are saturated fatty acids, with concentrations ranging from 11 to 15% and 4 to 7%, respectively [20]. The remaining fraction, constituting approximately 1% of the oil, consists of unsaponifiable substances. This fraction includes carotenoids, tocopherols, triterpene alcohols, sterols and xanthophylls [21]. The chemical composition of the oil determines its nutritional benefits.

The fatty acid composition of seven argan oil samples was analysed and the results (µg/g of sample) are summarised in Table 1. Based on the results, it can be concluded that argan oil is the oleic–linolenic type with linoleic acid (LA, C18:2(9Z,12Z), also known as vitamin F) comprising between 36% and 57%, and oleic acid (C18:1; 9Z) comprising between 20% and 47%. The analysis also revealed the presence of other fatty acids, including palmitic acid (PA, C16:0), stearic acid (SA, C18:0) and α-linolenic acid (ALA) (C18:3), with concentrations ranging from 5.5% to 14%, and 2.5% to 5%, respectively. However, the percentage of alpha-linolenic acid (ALA) (C18:3 (9Z,12Z,15Z)) in sample No. 7 was found to be 10%, which is noteworthy. The percentage content of the other 12 acids does not exceed 0.3% and is within the limits set by the 2005 Moroccan standard for virgin argan oils [20].

**Figure 1:**
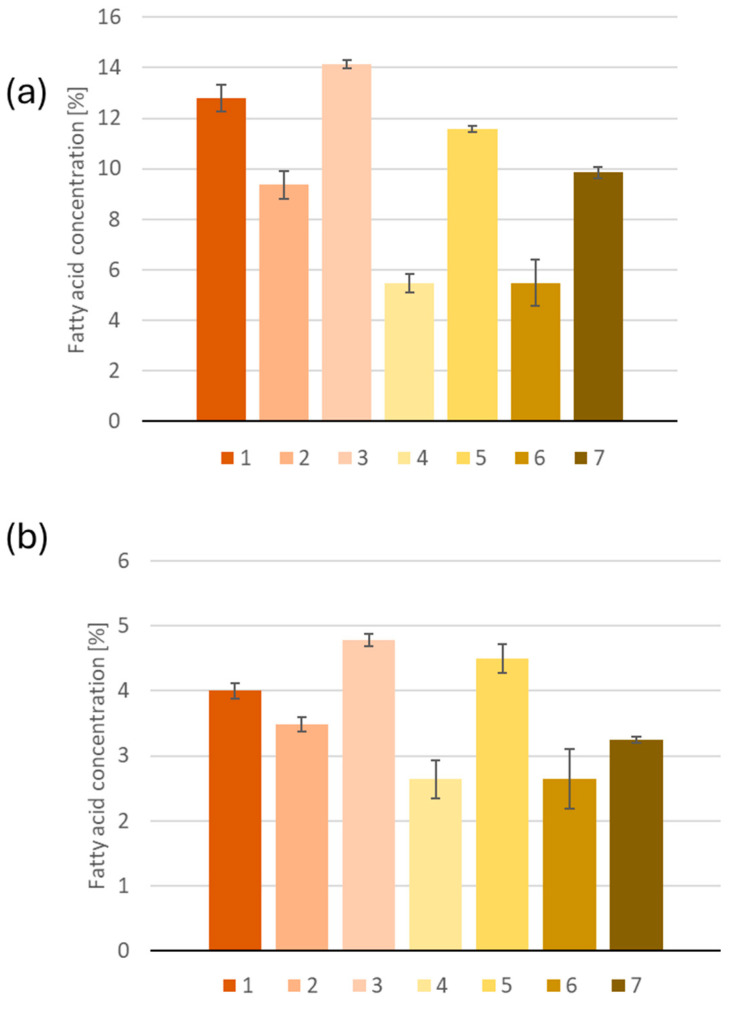
Composition of saturated fatty acids (**a**) palmitic acid (**b**) stearic acid in argan oil samples.

**Figure 2:**
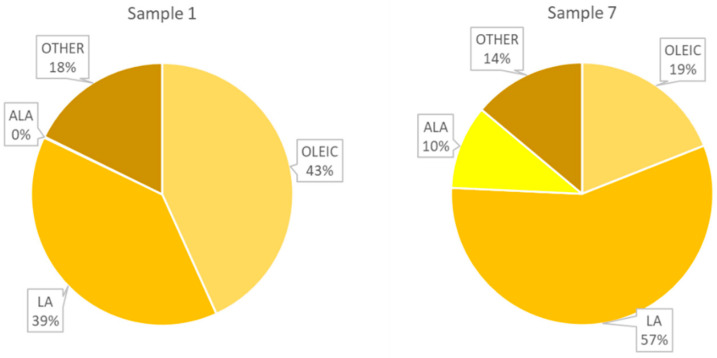
Comparison of the percentage of fatty acids in oils No. 1 and 7.

In addition to the acids identified in argan oil, the presence of long-chain C20:0 (average 0.15%), C20:1 (average 0.18%) and C22:0 (average 0.13%) acids were also noted. Notably, sample No. 3 was the only one without C15:1 and C14:2 fatty acids, while they were present in the other oils. It is noteworthy that oil No. 3 was the sole sample for which the determination of all 18 fatty acids was not feasible. The predominant acids identified in all oils were palmitic, stearic, oleic and linoleic (LA) acids. Palmitic and stearic acids are among the most prevalent saturated fatty acids, and it was observed that the concentration of palmitic acid exceeded that of stearic acid in all oils (Figure 1). In addition, oil No. 1 showed the highest concentration of palmitic acid (54.4 ± 2.2 mg/g sample), while oil No. 6 showed the lowest (16.1 ± 2.7 mg/g sample), demonstrating a normal ratio of more than three ALA, LA and oleic acid, which belong to the omega-3, -6 and -9 fatty acid families, respectively. The content of these fatty acids differed between the oils (Figure 2).

The highest quantity of oleic acid, which belongs to the omega-9 family, was found in oil No. 1 (183.5 ± 5.8 mg/g sample), while the lowest quantity was observed in oil No. 7 (75.9 ± 1.8 mg/g sample). These results were statistically different from the rest samples (*p* < 0.05).

Among the acids belonging to the EFA (essential unsaturated fatty acids) family, LA was the most prevalent. The highest concentration of this acid was observed in oils No. 7 and No. 2, (226.3 ± 6.0 and 204 ± 16 mg/g of sample, respectively). Conversely, the lowest concentration was observed in oil No. 5111.81 ± 0.42 mg/g of sample, which exhibited a concentration that was approximately 2-fold lower.

The greatest discrepancy in content was observed for ALA. The concentration of this fatty acid in oil No. 7 was 41.3 ± 1.5 mg/g sample, representing an 86- to 215-fold higher level compared to the other oils. Among oils 1–6, only oil No. 1 exhibited a notable elevation in this fatty acid content, reaching 495 ± 38 µg/g sample, which was 2-fold higher than the other oils.

**Figure 3:**
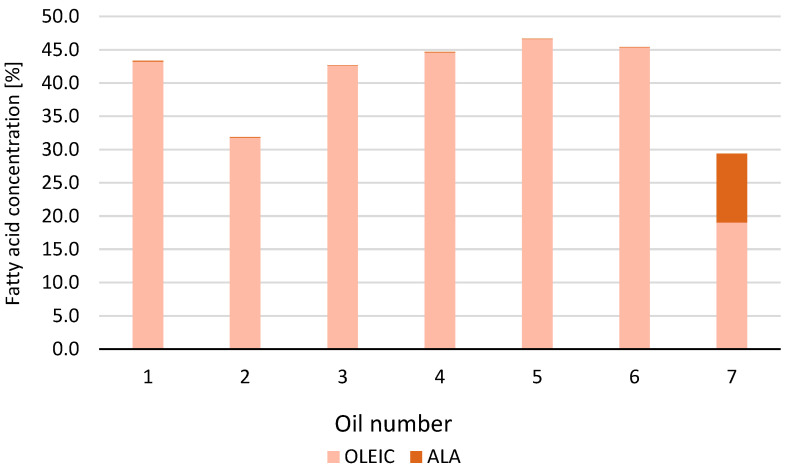
The ratio between oleic acid and α-linolenic acid (ALA) content.

A specific ratio of oleic acid and ALA content was also observed in oil samples (Figure 3). The oleic acid content was between 31 and 46%, the ALA content was below 0.2%. However, in oil No. 7, there was a higher oleic acid concentration (19.03%) with a significantly lower level of ALA (10.34%).

The methodology employed enabled the precise identification of individual fatty acids present in argan oil. Gas chromatography necessitates a meticulously prepared sample, which requires supplementary solvents and is inherently time-consuming. The processing and analysis of the results entail intricate calculations that are time-consuming, and the test material after measurement is no longer suitable for utilization in other analytical procedures.

The results demonstrate the variations in fatty acid profiles between argan oils derived from disparate sources. Each oil exhibited a distinctive composition, exhibiting similarities in quality but variable quantities. The differences between samples were frequently observed to be several-fold. As a natural product, variability in the proportions of individual fatty acids is expected. Natural resources vary due to differences in growing regions, plant varieties, year and time of harvest.

The observed differences in fatty acid content are statistically significant (*p* < 0.05), which may indicate a difference in quality. It is possible that these samples have undergone dilution or been subjected to processes that alter their composition. The discrepancies in the data may also be attributed to the disparate methods employed for oil extraction, the inherent quality of the raw material, the timing of the harvest and the age of the fruiting tree. The high content of linolenic acid (ALA) in oil No. 7 may be indicative of its freshness, suggesting that it has undergone minimal processing and storage. Alternatively, the production of oil No. 7 may have involved lower temperatures than the other oils, which could have prevented oxidation of the α-linolenic acid (ALA).

### 2.2. PCA Analysis

**Figure 4:**
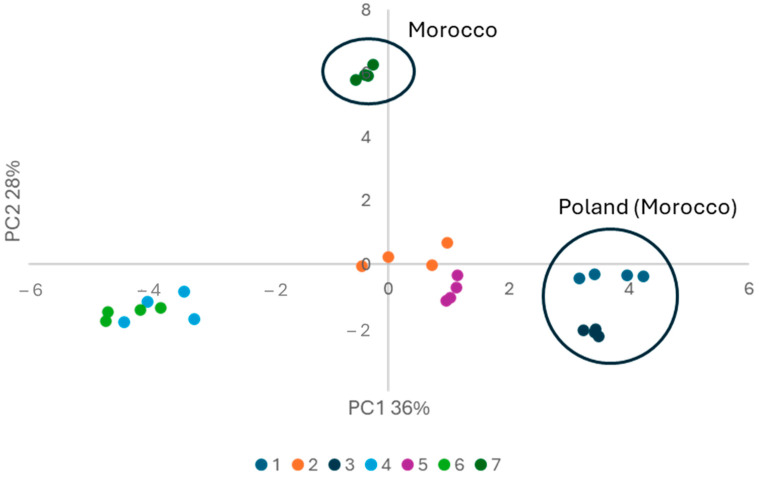
Score plot for oil samples from PCA analysis. Samples from Morocco are marked with circle (samples 1 and 3 from Morocco were bought in Poland).

To observe the grouping of samples and reduce the number of variables, PCA analysis was performed. Fatty acid composition from the GC experiment after normalization was used for further PCA calculations. Two new variables PC1 and PC2 explain 64% of total variance (respectively, 36% and 28%). Figure 4 shows the grouping of samples in the reduced variables coordinate system.

Points corresponding to samples 1–6 are grouped along the *X*-axis. Negative values of PC1 (samples 4, 6) correlate with a lower level of saturated fatty acids (C10:0, C12:0, C14:0, C15:0, C16:0, C17:0, C18:0, C20:0), while positive values mean a higher level of these fatty acids. It can be seen that in the examination of fatty acid composition, the number of analysed fatty acids may be reduced only to the determination of palmitic acid (C16:0). Palmitic acid is a main saturated fatty acid in argan oil and changes in saturated fatty acid concentration is reflected in the level of palmitic acid. PC2 variable shows changes in unsaturated fatty acids. The greatest impact on points shifting along the *Y*-axis on the graph is the concentration of α-linolenic acid. The concentration of ALA in sample 7 is 10 times higher than in other samples. There are also statistically significant differences in the composition of other unsaturated fatty acids, but the differences are much lower, so they do not have as great an impact on the point shifting along the *Y*-axis.

Additionally, it can be seen, that samples with the same description of origin have similar composition of fatty acids. A description of country of origin/purchase can be found in Material and Methods section. Samples bought in Poland, and obtained from Morocco (due to labelling) are grouped in the 4th quarter (samples 1 and 3). The sample bought in Morocco is separated from other samples. It has different fatty acid compositions, especially in unsaturated fatty acids. It is an untypical composition for argan oil and may indicate falsification of the oil.

### 2.3. ^1^H NMR Study

To ascertain the fatty acid composition of the tested argan oils, they were subjected to one-dimensional proton nuclear magnetic resonance (^1^H NMR) analysis. The resulting spectra were processed using Mestre nova 6.0.2 software, and the individual signals were then assigned to protons from the different functional groups present in the acids. The chemical shift values for the proton groups are presented in Table 2.

**Figure 5:**
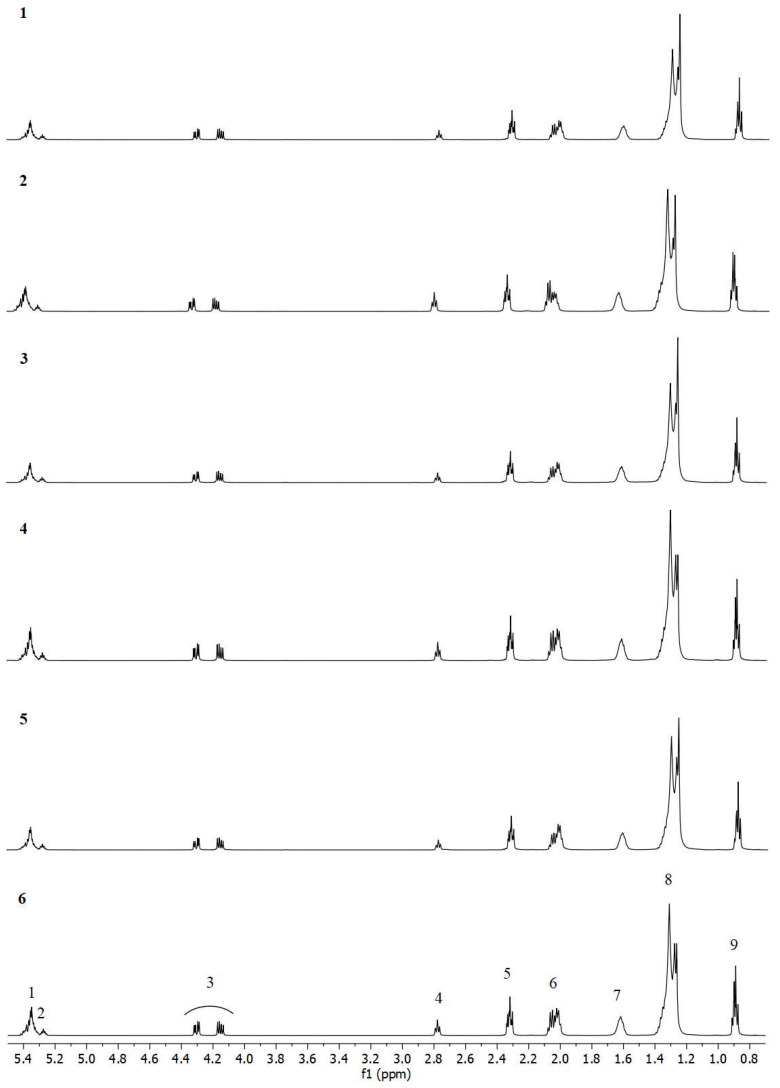
^1^H NMR spectra in CDCl_3_ of oil No. 1–6. The numbers of individual proton groups from 1 to 9 are presented in Table 2.

The spectra for the six oil samples subjected to analysis are presented in Figure 5. The spectra displayed in Figure 5 illustrate the presence of nine discrete, complex signals (multiplets) spanning a range of 0.8 ppm to 5.5 ppm. The ^1^H NMR spectra for the six samples exhibit no notable differences in their visual representation. At a chemical shift value of δ 5.37–5.32 ppm, signals are observed from protons bonded to carbon atoms that are joined by a -CH=CH- double bond (signal No. 1). This configuration is indicative of monounsaturated and polyunsaturated fatty acids, which are present in all the oils under examination. The multiplet observed at δ 2.09–1.99 ppm originates from the protons of the methylene group linked to the vinyl group (-CH_2_-CH=CH-) (signal No. 6).

Signals occurring at chemical shift values of δ 5.29–5.25 ppm originate from the protons of the >CHOCOR grouping (signal No. 2), while those at δ 4.32–4.28 and δ 4.18–4.13 ppm are attributed to the protons of the methylene group of the -CH_2_OCOR system (signal No. 3). The range of chemical shift values observed for these resonances is characteristic of triacylglycerols. The ^1^H NMR spectra are described in Table 1. Signals from LA and ALA are also discernible, emanating from the methine group of the =CH-CH_2_-CH= system. A signal at chemical shift values δ 2.80 is observed for protons of the methine group. Signals at chemical shift values of −2.75 ppm (signal No. 4) and of the methylene group at δ 0.91–0.87 ppm in the -CH_2_-CH_3_ grouping (signal No. 9) are also observed. The signal at the shift value of δ 2.34–2.29 ppm is derived from protons of the methylene group that are linked by oxygen to the carbonyl carbon atom, -OCO-CH_2_-. This is indicated as signal No. 5. Such groupings are specific to acyl chains in unsaturated fatty acids. Signal No. 7, observed at a chemical shift of δ 1.65–1.58 ppm, may originate from squalene (an unsaturated hydrocarbon) or from a methylene group from the -OCO-CH_2_- acyl chain. The presence of the latter is confirmed by the presence of signal 8, observed at a chemical shift of 1.40–1.23 ppm -(CH_2_)_n_-, which is specific for acyl chains.

**Figure 6:**
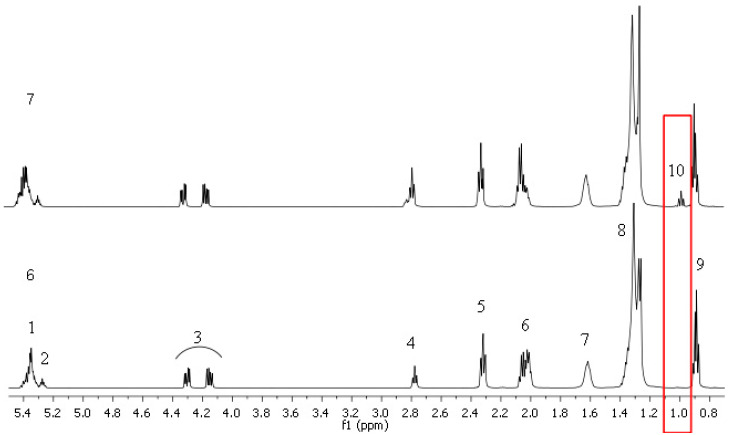
^1^H NMR spectra in CDCl_3_ of oil No. 6–7. The numbers of individual proton groups from 1 to 10 are presented in Table 2.

The ^1^H NMR spectrum of oil sample No. 7 exhibits the presence of an additional signal (No. 10) at values of δ 1.00–0.96 ppm, which is indicative of the -CH=CH-CH_2_-CH_3_ methyl group, a characteristic feature of linolenic acid (Figure 6).

**Figure 7:**
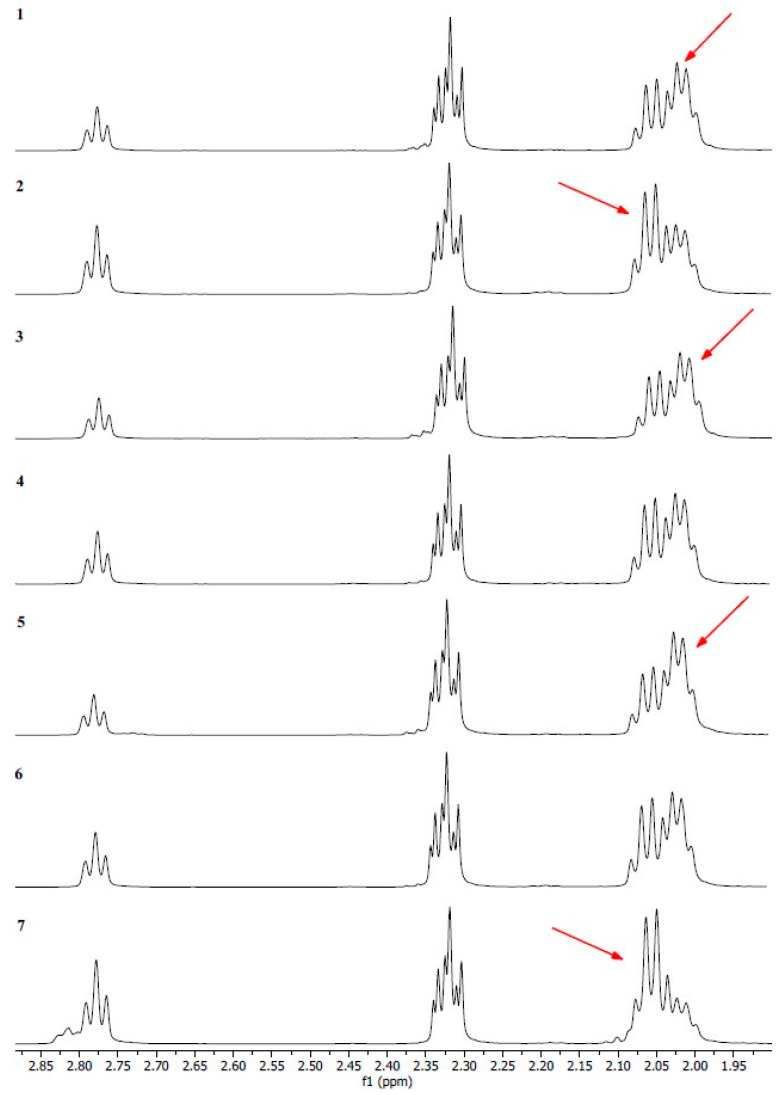
^1^H NMR spectra in CDCl_3_ of oil No. 1–7.

Notwithstanding the identical signals identified in oils 1–7, notable discrepancies in the intensity of signal 6 are evident (Figure 7). It was observed that the signals exhibited a high degree of similarity for oils 1, 3 and 5, with the signals in the δ 2.05–2.00 ppm range displaying the greatest intensity. Significant signals were observed in the δ 2.10–2.05 ppm range for oils 2 and 7, which are derived from protons present in mono- and polyunsaturated fatty acids. Oils 4 and 6 exhibited similar signal intensities throughout the δ 2.10–2.00 ppm range.

Moreover, the spectra of oils 2 and 7 exhibited distinctive signals in the range δ 2.80–2.75 ppm, which were more intense than those observed in the other oil samples.

Given the observed differences in intensity, a quantitative analysis was conducted. The characteristic signals in the ^1^H NMR spectra were subjected to integration based on signal intensity in MestreNova 6.0.2 software, from which the ratios of the individual fatty acids were then calculated using formulas. The resulting fatty acid ratios are presented in Table 3.

The results demonstrate a deficiency in ALA acid content in the argan oils included in samples 1–6 (Table 3). It should be noted that the results presented are specific to oil number Sample 7, which exhibited a content of approximately 3.5% of the acid in question.

The LA contents exhibited considerable variation between the oils. The highest content was observed in oil No. 7 (27.97%), while the lowest was noted in oil No. 3 (13.03%), which was approximately 2-fold lower. Significant differences were also evident in oleic acid contents between oils. The highest contents were observed in oils No. 4–6 (71.24–74.09%), while the lowest was observed in oil No. 7 (55.05%). A correlation can be observed between lower oleic acid content with concomitant differences in ALA content.

The MUFA contents were found to be high for all oils. The lowest values in terms of MUFA content were observed in samples 2 and 7. Conversely, the reverse was true for PUFA, where samples 2 and 7 oils exhibited higher polyunsaturated (PUFA) than monounsaturated (MUFA) contents. In addition, the highest content of saturated fatty acids was observed in oil 3, while the lowest amount was found in oils 4 and 6.

**Figure 8:**
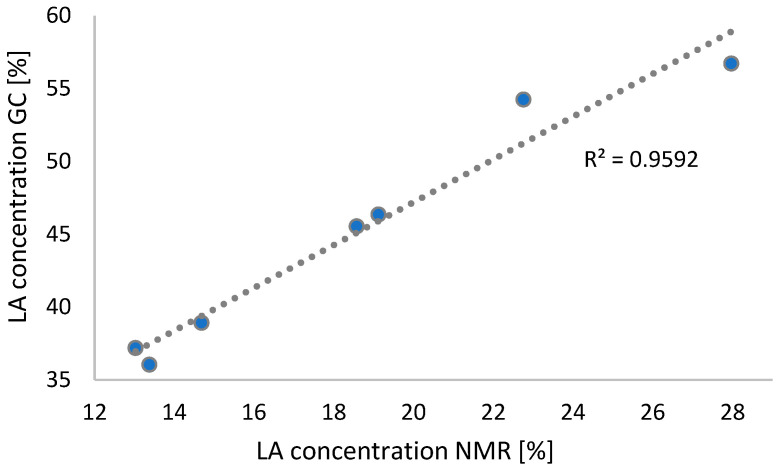
Comparison of LA content by GC and ^1^H NMR methods.

The outcomes of the quantitative analysis of the selected fatty acid, LA, as observed through the utilisation of ^1^H NMR spectroscopy, were then compared with those obtained through the conventional methodology of gas chromatography. The linear relationship between the two sets of data is illustrated in Figure 8, with the R^2^ coefficient value being 0.9592. The correlation coefficient thus obtained indicates a high degree of correlation and serves to confirm the validity of employing these two methods in a complementary manner.

The ^1^H NMR method proved an expeditious and perspicacious means of identifying the characteristic signals indicative of the presence of discrete components (fatty acids) of argan oil. The analysis did not necessitate the preparation of a special sample. The processing of the obtained spectra did not require a significant amount of time (the recording time was a few minutes). The quantity of fatty acids identified in the ^1^H NMR spectra is considerably lower in comparison to the results obtained by chromatographic (GC) analysis. Nevertheless, it allows for a qualitative assessment of the material under study.

A comparative analysis of the ^1^H NMR spectra of the oils under examination revealed notable discrepancies in their compositional profiles. The most significant discrepancy was observed in the presence of ALA acid exclusively in oil number seven. It is possible that external factors may have influenced the oils tested (Nos. 1–6) in a way that resulted in the oxidation of ALA acid to less saturated or saturated fatty acids. Furthermore, differences in the intensity of signals 4 and 6 were observed in oils No. 2 and 7, which are responsible for the differences in MUFA and PUFA acid content. A quantitative analysis corroborated this finding, revealing that only these oils exhibited a higher proportion of PUFA relative to MUFA.

While the same nine signals were observed in all oils, significant differences were noted in the intensity of the individual signals. Quantitative analysis using ^1^H NMR spectra confirmed the highest unsaturated fatty acid contents in oils 4 and 6, which were also recorded by the standard GC method. The LA acid content determined by ^1^H NMR exhibited a high degree of correlation with the results obtained by GC, demonstrating the similar selectivity of the ^1^H NMR method with GC.

### 2.4. UV-Vis Study

**Figure 9:**
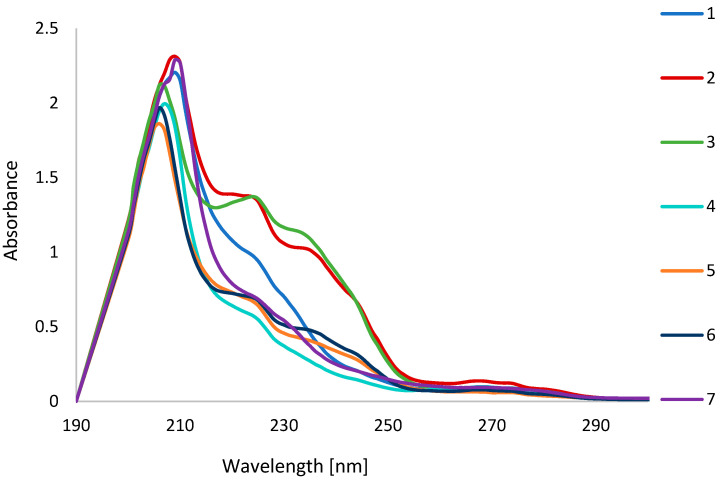
The UV-vis spectra of oils.

A further method for evaluating the composition of argan oils is the measurement of ultraviolet–visible (UV-Vis) absorbance. Based on the absorbance measurements of the individual oils in the range of 200–400 nm, the spectra were plotted (Figure 9). It is evident that there are differences in the absorbance intensity within the wavelength range λ 220–230 nm, which is indicative of the degree of polyunsaturated fatty acid dienes formed during the various transformations of the vegetable oil. The highest values in this wavelength range were exhibited by oils 2 and 3, which may suggest a shorter shelf life compared to the other oils. The other oils demonstrated similar absorbance values at these wavelengths, which may indicate a greater degree of freshness.

The application of UV-Vis spectroscopy enables the differentiation of the extent of transformation occurring in individual vegetable oils, which may serve to indicate the relative freshness of the oil. However, this method does not permit the identification of the individual components of a vegetable oil or the confirmation of its authenticity. The assessment of the shelf life of vegetable oils can be achieved through the utilization of the UV-Vis method.

## 3. Materials and Methods

The study employed seven samples of cosmetic argan oil, procured from disparate producers. The samples were procured from commercial establishments in Poland, Turkey and Morocco between November 2022 and November 2023. As indicated by the manufacturers, the products were composed exclusively of argan oil (100%). They were stored in their original packaging or in protected Eppendorf’s at 4 °C. The composition and country of origin of the samples are presented in Table 4.

### 3.1. Determination of Fatty Acid Content with GC-MS Technique

Samples of seven different brands of argan oil were purchased from various sources. As indicated by the manufacturers, the products were composed exclusively of argan oil (100%). They were stored in their original packaging or in protected Eppendorfs at 4 °C. All analyses were performed from fresh oil in four replicates.

The standard analytical methods used for the quality determination of oils have been applied [22]. The tests were conducted at the Department of Animal Nutrition of The Kielanowski Institute of Animal Physiology and Nutrition, Polish Academy of Sciences.

As fatty acid methyl esters (FAMEs), the fatty acid (FA) profiles of the oil samples were determined using capillary gas chromatography coupled with mass spectrometry (GC-MS). The chromatographic analysis required that the FAs be preserved using mild alkali- and acid-catalysed methylation procedures, which were performed according to the procedure proposed by Czauderna’s team [23] and based on the research experience of previous ones [22], and, in the next step, quantified using a gas chromatograph coupled to a mass spectrometer (Shimadzu GC-MS-QP2010 Plus EI, Tokyo, Japan). The chromatographic system was equipped with a BPX70 fused silica column (120 m × 0.25 mm i.d. × 0.25 µm layer thickness; Phenomenex, Torrance, CA, USA), a quadruple mass detector (Model 5973 N) and an injection port. Nonadecanoic acid (99%, Sigma, St. Louis, MO, USA) was used as an internal standard (IS). The identification of FAME was based on electron ionisation spectra of FAME and compared with authentic FAME standards (Supelco 37 Component FAME Mix, Sigma, St. Louis, MO, USA), the c9t11c13C18:3-PA methyl ester standard (Methyl punicate, Matreya LCC, State College, PA, USA), the c9t11t13C18:3-α-eleostearic methyl ester standard ESA (Larodan Fine Chemicals, Solna, Sweden) and the NIST 2007 reference library of mass spectra (National Institute of Standards and Technology, Gaithersburg, MD, USA). Analyses were performed for the prepared four parallel samples for each product. All FAME analyses performed were based on total ion current chromatograms and/or selected ion monitoring chromatograms. The results were expressed as µg/g oil or mg/g oil.

### 3.2. ^1^H NMR

A one-dimensional nuclear magnetic resonance (NMR) spectroscopy analysis was conducted on all seven oil samples. The spectra were recorded on an Avance Neo 400 NMR spectrometer and an Avance III 500 NMR spectrometer (11.7 T) from Bruker at the Centre for Molecular and Macromolecular Studies, Polish Academy of Sciences, Lodz, Poland, in the Environmental Physicochemical Research Laboratory of Organic Compounds and Polymers. For analysis, each oil was dissolved in deuterated chloroform (CDCl_3_, 99.5%, Armar Chemicals). The resulting spectra were processed using Mestrenova 6.0.2 software, and the data were used for qualitative and quantitative analysis of the selected fatty acids present in the analysed products.

Proton signals were identified through a review of the available literature. The quantitative content of fatty acids was determined, including ALA (alpha-linolenic acid), LA (linoleic acid), OLEIC (oleic acid), TOTUNSAT (unsaturated fatty acids), TOTSAT (saturated fatty acids) and MUFA (monounsaturated fatty acids). The content of polyunsaturated fatty acids (PUFAs) was calculated using the formulae [16] by integrating the individual signals (signal numbering according to Table 2), which were determined using Mestrenova 6.0.2 software.

(1) $$ALA=\frac{I_{10}}{I_{10}+I_{9}}$$

(2) $${TOT}_{UNSAT}=OLEIC+LA+ALA$$

(3) $$LA=\frac{\left(3\cdot I_{4}-4\cdot I_{10}\right)}{2\cdot ({I_{9}+I}_{10})}$$

(4) $${TOT}_{SAT}=100-{TOT}_{UNSAT}$$

(5) $$OLEIC=\frac{I_{6}}{4\cdot k}-ALA-LA$$

(6) $$MUFA=\frac{4I_{10}+{3I}_{6}-{6I}_{4}}{{6\cdot I}_{5}}$$

(7) $$k=\frac{I_{5}}{2}$$

(8) $$PUFA=\frac{{3I}_{4}-2I_{10}}{{3\cdot I}_{5}}$$

### 3.3. UV-Vis Spectroscopy

The seven oils were weighed on an analytical balance (with an average sample weight of approximately 55 mg) and then dissolved in an organic solvent (n-hexane, with a volume of 5 mL). The measurements were conducted using a 400-fold dilution. The determination was carried out on three occasions within the wavelength range of 200 to 400 nm, utilising a spectrophotometer (UV-1900i UV-Vis, Shimadzu, Tokyo, Japan).

### 3.4. Statistical Analysis

Results of individual FA content and sums of FA of the different oils were analysed by one-way ANOVA followed by the Tuckey test to compare significant variations between means (*p* < 0.05). Moreover, in order to verify the capacity of the FA analysis as a tool for oils characterization a multivariate discriminant analysis was performed. The basic purpose of discriminant analysis is to estimate the relationship between a single categorical dependent variable (the type of oil in this case) and a set of quantitative independent variables (the percentage contents of the fatty acids), since the relationships between oils are affected by all the variables and only analysing the combination effects by multivariate methods similarities and differences between oils can be established effectively. This analysis can determine which of the independent variables accounts the most for the differences in the average score profiles of the different oils. Moreover, the graph representation of this analysis allows for assessing the similarity of the oils by their FA composition. All the statistical analyses were carried out using the Statistica 13.

## 4. Conclusions

Argan oil is a valuable raw material, sourced in only one region in the world. It is necessary to verify the authenticity of argan oil. It is essential to have the necessary analytical tools for controlling the origin and the authenticity of argan oil. Such measures can protect producers and consumers from fraudulent activity.

The methodologies employed permitted the differentiation of the argan oils under examination to varying degrees. The most accurate qualitative and quantitative method is gas chromatography, which permitted the identification of 18 distinct fatty acids. However, this method necessitates the implementation of intricate procedures for the preparation of the sample and the processing of the resulting data. A less accurate quantitative method, but an equally efficacious technique for the identification of fatty acids, was the ^1^H NMR method. This method enabled the swift and effective differentiation between the oils under examination, obviating the necessity for bespoke sample preparation. The results of the two methods exhibited a high degree of correlation, thereby supporting the use of ^1^H NMR as a complementary method for the assessment of argan oil authenticity, particularly in the context of rapid screening diagnostics.

The UV-Vis method proved unable to identify the fatty acids present in argan oils. The apparent differences in the spectra obtained may suggest differences in the composition of the oils, but this cannot be confirmed with certainty. The UV-Vis method may allow an assessment of freshness or storage quality, which is also a factor in the quality of the oils.

The findings of the study indicated that the most efficacious oil was No. 7, which was procured from a commercial establishment in Morocco. Although the oil did not contain the highest content of individual fatty acids (ALA, LA, oleic, stearic and palmitic acids), it was the only oil tested that contained all the aforementioned fatty acids in its composition, including ALA. The most notable oil was also No. 1, which exhibited the highest concentration of individual acids, contained 2-fold the amount of ALA acid compared to the other five oils, and had a relatively low price point. The specific extinction test also corroborated the superior freshness and shelf life of these two oils.

However, it is not possible to clearly identify which oil was of the poorest quality. Only for oil No. 3 could all 18 fatty acids not be determined; it contained the highest content of saturated fatty acids and was most exposed to poor storage conditions. In contrast, oil No. 6 showed the lowest content of selected fatty acids and the lowest content of saturated fatty acids. In contrast, oil No. 2 had the fattest oxidation processes.

## Figures and Tables

**Table 1:** Mean fatty acid content of the individual oils, letters show statistically significant differences (post hoc Tukey test, *p* < 0.05).

	1	2	3	4	5	6	7
FA [ug/g of Sample]	Mean ± SD	Mean ± SD	Mean ± SD	Mean ± SD	Mean ± SD	Mean ± SD	Mean ± SD
C10:0	36.1 ^a,b^ ± 5.9	43 ^a,b^ ± 10	48.2 ^b^ ± 8.0	35.7 ^a,b^ ± 9.9	39.6 ^a,b^ ± 3.5	27.0 ^a^ ± 7.0	37.0 ^a,b^ ± 4.0
C12:0	58 ^a,b^ ± 12	73 ^b^ ± 10	62.0 ^a,b^ ± 8.1	52.4 ^a,b^ ± 8.9	54 ^a,b^ ± 11	48.2 ^a^ ± 3.5	52.5 ^a,b^ ± 7.8
C14:0	789 ^a^ ± 39	557 ^b^ ± 40	708 ^c^ ± 16	311 ^d,e^ ± 29	535 ^b^ ± 28	272 ^e^ ± 23	347 ^d^ ± 30
C15:0	234 ^a^ ± 22	138 ^b^ ± 31	202.3 ^a,c^ ± 6.1	65 ^d^ ± 14	168 ^b,c^ ± 11	50.5 ^d^ ± 7.2	73.2 ^d^ ± 8.4
C16:0 [mg/g of sample] *	**54.4 ^a^ ± 2.2**	**35.2 ^b^ ± 2.1**	**47.3 ^c^ ± 0.5**	**18.0 ^d^ ± 1.3**	**35.9 ^b,e^ ± 0.4**	**16.1 ^d^ ± 2.7**	**39.30 ^e^ ± 0.84**
C17:0	269 ^a^ ± 31	193 ^b^ ± 27	267 ^a^ ± 18	116 ^c^ ± 20	223 ^a,b^ ± 23	97 ^c^ ± 15	369 ^d^± 18
C18:0 [mg/g of sample] *	**16.99 ^a^ ± 0.48**	**13.10 ^b^ ± 0.42**	**16.00 ^a^ ± 0.31**	**8.68 ^c^ ± 0.94**	**13.9 ^b^ ± 0.7**	**7.7 ^c^ ± 1.3**	**12.94 ^b^ ± 0.20**
C20:0	569 ^a,b^ ± 29	475 ^a^ ± 41	557 ^a,b^ ± 46	363 ^c^ ± 32	576 ^a,b^ ± 47	313 ^c^ ± 57	605 ^b^ ± 67
C22:0	117 ^a^ ± 19	504 ^b^ ± 23	104.2 ^a^ ± 9.2	744 ^c^ ± 61	262 ^d^ ± 12	649 ^c^ ± 61	345 ^d^ ± 72
C15:1	46.7 ^a^ ± 8.2	31.9 ^a,b^ ± 5.0	-	14 ^c,d^ ± 16	38.1 ^a,b^ ± 7.2	21.6 ^b,d^ ± 5.9	78.0 ^e^ ± 2.7
C16:1	764 ^a^ ± 46	1004 ^b^ ± 119	1160 ^c^ ± 97	578 ^d^ ± 28	729 ^a^ ± 15	648 ^a,d^ ± 82	548 ^d^ ± 28
C17:1	90 ^a,b^ ± 17	105 ^a,b^ ± 17	73.7 ^a^ ± 7.9	117.0 ^b^ ± 9.6	85 ^a^ ± 13	86 ^a,b^ ± 19	180.4 ^c^ ± 7.3
C18:1(9Z) [mg/g of sample] *	**183.5 ^a^ ± 5.8**	**119.4 ^b^ ± 7.0**	**142.6 ^c^ ± 3.1**	**146.8 ^c^ ± 9.7**	**144.6 ^c^ ± 3.3**	**133 ^b,c^ ± 20**	**75.9 ^d^ ± 1.8**
C20:1	1007 ^a^ ± 46	605 ^b^ ± 65	851 ^c^ ± 55	398 ^d^ ± 47	831 ^c^ ± 68	367 ^d^ ± 78	414 ^d^ ± 31
C14:2	33 ^a,b^ ± 12	42 ^a^ ± 10	-	15 ^b^ ± 18	33.2 ^a,b^ ± 4.8	30.3 ^a,b^ ± 9.3	48.7 ^a^ ± 5.4
**C18** **:2(9Z,12Z) [mg/g of sample]**	**165.3 ^a^ ± 5.3**	**204 ^b^ ± 16**	**124.4 ^c^ ± 2.4**	**152.5 ^a,d^ ± 7.7**	**111.81 ^c^ ± 0.42**	**133 ^c,d^ ± 22**	**226.3 ^b^ ± 6.0**
C18:3(9Z,12Z,15Z) *	**495 ^a^ ± 38**	**295 ^a^ ± 34**	**221.9 ^a^ ± 8.9**	**258 ^a^ ± 15**	**159.7 ^a^ ± 5.6**	**212 ^a^ ± 41**	**41281 ^b^ ± 1513**
C20:2	51 ^a^ ± 12	136 ^a,b^ ± 34	44.4 ^a,b^ ± 3.8	54 ^c^ ± 11	161 ^b^ ± 17	49 ^c^ ± 19	247 ^d^ ± 33

* concentrations expressed in mg/g of sample

**Table 2:** Chemical shift values of proton signals.

Signal	^1^H δ ppm	Group of Labelled Protons H	Functional Group
1	5.37–5.32	-CH=CH-	Mono- and polyunsaturated fatty acids Olefinic protons
2	5.29–5.25	>CHOCOR	Triacylglycerols
3	4.32–4.28 4.18–4.13	-CH_2_OCOR	Triacylglycerols
4	2.80–2.75	=CH-CH_2_-CH=	Linoleic acid Linolenic acid
5	2.34–2.29	-OCO-CH_2_-	Acyl chains in unsaturated fatty acids
6	2.09–1.99	-CH_2_-CH=CH-	Mono- and polyunsaturated fatty acids
7	1.65–1.58	SQUA	Squalene
		–OCO-CH_2_-CH_2_-	Acyl chains
8	1.40–1.23	-(CH_2_)_n_-	Acyl chains
9	0.91–0.89 0.89–0.87	-CH_2_-CH_3_	Linoleic acid Linolenic acid
10	1.00–0.96	-CH=CH-CH_2_-CH_3_	Linolenic acid

**Table 3:** Calculated percentages of individual fatty acids: ALA—linolenic acid, LA—linoleic acid, OLEIC—oleic acid, TOT_UNSAT_—unsaturated fatty acid, TOT_SAT_—saturated fatty acid, MUFA—monounsaturated fatty acid, PUFA—polyunsaturated fatty acid.

Number	ALA	LA	OLEIC	TOT UNSAT	TOT SAT	MUFA	PUFA
	[%]						
1	0.00	14.68	68.27	82.96	17.04	53.02	29.94
2	0.00	22.76	63.21	85.97	14.03	39.89	46.08
3	0.00	13.03	62.25	75.28	24.72	49.07	26.21
4	0.00	19.12	74.09	93.21	6.79	55.35	37.86
5	0.00	13.37	71.24	84.61	15.39	57.64	26.97
6	0.00	18.58	73.73	92.31	7.69	55.22	37.09
7	3.56	27.97	55.05	86.59	13.41	24.09	62.50

**Table 4:** Numbers, origin and composition of tasted cosmetic raw materials argan oil.

Number	Country of Purchase (Origin on Label)	Composition According to Manufacturer
1	Poland (Morocco)	100% argan oil
2	Poland	100% argan oil
3	Poland (Morocco)	100% argan oil
4	Poland	100% argan oil
5	Turkey	100% argan oil
6	Poland	100% argan oil
7	Morocco (Morocco)	100% argan oil

## Data Availability

The original contributions presented in this study are included in the article. Further inquiries can be directed to the corresponding author.
